# Emergence of Nontrivial Spin Textures in Frustrated Van Der Waals Ferromagnets

**DOI:** 10.3390/nano11071770

**Published:** 2021-07-07

**Authors:** Aniekan Magnus Ukpong

**Affiliations:** Theoretical and Computational Condensed Matter and Materials Physics Group, School of Chemistry and Physics, University of KwaZulu-Natal, Pietermaritzburg 3201, South Africa; ukponga@ukzn.ac.za; Tel.: +27-33-260-5875

**Keywords:** spin current, van der Waals ferromagnets, magnetic skyrmion, spin Hall effect

## Abstract

In this work, first principles ground state calculations are combined with the dynamic evolution of a classical spin Hamiltonian to study the metamagnetic transitions associated with the field dependence of magnetic properties in frustrated van der Waals ferromagnets. Dynamically stabilized spin textures are obtained relative to the direction of spin quantization as stochastic solutions of the Landau–Lifshitz–Gilbert–Slonczewski equation under the flow of the spin current. By explicitly considering the spin signatures that arise from geometrical frustrations at interfaces, we may observe the emergence of a magnetic skyrmion spin texture and characterize the formation under competing internal fields. The analysis of coercivity and magnetic hysteresis reveals a dynamic switch from a soft to hard magnetic configuration when considering the spin Hall effect on the skyrmion. It is found that heavy metals in capped multilayer heterostructure stacks host field-tunable spiral skyrmions that could serve as unique channels for carrier transport. The results are discussed to show the possibility of using dynamically switchable magnetic bits to read and write data without the need for a spin transfer torque. These results offer insight to the spin transport signatures that dynamically arise from metamagnetic transitions in spintronic devices.

## 1. Introduction

Improved microelectronic reliability in non-volatile random access memory, increased data storage density, enhanced robustness of the magnetic state of stored information, and stability against external perturbations underscore all contemporary efforts to develop new materials for high-density magnetic storage devices. For instance, current spin-based memory technologies require the on-chip integration of magnetic bits for ultrafast-switching with significantly reduced power requirements; however, owing to the poor scalability of the conventional method of electrical spin injection, alternative methods to generate, transport, and detect pure spin currents are currently being pursued. Strategies that are based on material-dependent intrinsic fields are of special interest, such as those of Heisenberg exchange [1], Dzyaloshinskii–Moriya exchange [2], spin–orbit coupling (SOC) [3], and defect-induced *d*^0^ magnetism [4]. This has led to suggestions of considering a magnetic skyrmion (MS) as an information carrier. As such, concerted attempts are being made to understand the formation, stability, and tuning capabilities of magnetic skyrmions. For instance, initial observations of persistent spin helices in intrinsically nonmagnetic (NM) quantum-well semiconductors [5,6,7] have stimulated interest in using the internal effective fields of materials to dynamically create robust spin textures.

A MS is a topologically protected spin texture which is stabilized by the Dzyaloshinskii–Moriya interactions (DMIs) [8,9,10,11]. Other common examples include the Neel-type (i.e., hedgehog-like) skyrmion, Bloch-type (i.e., combed hedgehog) skyrmion and antiferromagnetic (AFM) skyrmion. Persistent symmetry-enforced spin textures are hosted in non-centrosymmetric bulk materials [12], whereas recent observations of charge density waves and frustrated spins in monolayer VSe_2_ have been attributed to spin frustration [13]. Exotic spin textures are observed in the trilayer heterostructure of Ta(5 nm)/Co_20_Fe_60_B_20_(CoFeB)(1.1 nm)/TaO_x_(3 nm) as magnetic bubbles. The latter are polar skyrmions whose spin textures consist of a dipolar topological charge [14]. Polar skyrmions are also quasiparticles, i.e., small magnetic swirls, but they possess reversible electrical properties. Such magnetic bubbles have also been observed in (PbTiO_3_)_n_/(SrTiO_3_)_n_ superlattices [15], suggesting that aside from non-centrosymmetric ferromagnets, engineered multilayer heterostructures [16] could also host nontrivial MS states in the presence of SOC.

In addition, the magnitudes of spin currents [17] generated by the direct spin Hall effect (SHE) [18,19], Rashba–Edelstein effect [20,21], spin pumping [22,23], and nonlocal electrical spin injection from a ferromagnetic (FM) layer [24,25] depend on the degree of spin scattering in the heavy metal (HM) layer. Crucially, the spin scattering also depends on the strength of the SOC in the HM layer. Besides, during the flow of spin current, the spin–orbit torque (SOT) in HM/FM interfaces also offers an effective strategy for low-power switching [26,27,28,29]. Thin multilayered films host chiral spin textures, such as skyrmions, when the interfacial DMI is mediated by the SOC of a paramagnetic HM in a system that lack structural inversion symmetry. The resulting magnetic frustration provides ample opportunities for the formation of nontrivial soliton-like phases in solids. The prerequisite for analyzing spin texture in magnetic solitons is to characterize the formation and stability under realistic device conditions. Skyrmions are the smallest non-trivial local structures that possess a distinct spin texture with a fixed chirality. Their quasi-particle nature attracts fundamental scientific interest [30], as skyrmion-based insights promise practical utility in microelectronic devices [31]. The recent observation of Bloch-type skyrmions in Gd_2_PdSi_3_, a centrosymmetric magnet with a triangular lattice, further underscores the role of SOC in geometrically frustrated magnets [32].

Our recent studies of carrier transport in a multilayer van der Waals system of Fe(110)/hBN/*M* stacks have revealed that an applied external axial field induces dynamic spin-sensitive signatures in the spin transport phase [33]. In this case, hBN denotes the hexagonal boron nitride monolayer that is incorporated in the tunnel barrier region and *M* = V, Co, or Ni. Nonetheless, the spin texture of this carrier transport phase is still not clear. In this work, we unravel the formation of the MS spin texture under SHE conditions when a single FM/hBN/HM interface is retained in the heterostructure, wherein the *M* species is replaced with HM species, Pt, Pd, and Ta. Because of the lack of structural inversion symmetry in a multilayer stack and the inherent non-magnetic characteristics of both hBN and HM species, a nonvanishing magnetization is obtained in the Heisenberg spin model of the multilayer stacks when at a ground state. An Ising-like spin ice model is used to represent the resultant magnetization of the stack in terms of site-resolved magnetic spin moments. In addition, the intrinsic broken time reversal symmetry of the electron states that arise from the intrinsic SOC in the HM layer makes the stacks potential hosts for complex spin textures. The interfacial spin signatures that arise from spin injection at the heterobilayer interfaces are considered explicitly here.

Herein, first principles calculations of the ground state magnetization in Fe/hBN/HM multilayer ferromagnets are combined with a consideration of the classical dynamics of atomistic spinning to study the spin textures that arise from metamagnetic transitions in van der Waals ferromagnetic stacks. An Ising-like spin ice model captures the field-dependent evolution of the unique filtering of spins into spatially localized domains with upward (↑↑) and downward (↓↓) textures in the presence of SOT and favors the emergence of the MS spin texture in the transport phase. The calculated hysteresis loops and the coercive fields reveal that the ground state is dynamically switched from a soft to hard ferromagnetic state when under a spin Hall regime. Under an applied external magnetic field, we observe a continuous change in the spin texture that is analogous to the instability of skyrmions in weak magnetic fields. The low magnetic field instability manifests as a change in skyrmion shape from spherical to rod-like in terms of the local structure. We conclude that HM-capped van der Waals multilayer stacks host field-tunable spiral-type skyrmions [34,35], and that these could serve as a collective transport mode for field-dependent carrier transport. The results are discussed to illustrate the possibility of using a dynamically switchable magnetic bit to read or write data in spintronic applications without the need for a spin transfer torque (STT).

## 2. Theoretical and Computational Methods

### 2.1. Frustrated Van Der Waals Ferromagnets

Frustrated van der Waals magnets were modeled as Fe/hBN/HM heterostructures (HM = Pt, Pd, and Ta) here. These are like Fe/hBN/M stacks [33,36,37], except that M is replaced with a HM species. In these stacks, intrinsic geometrical frustration arises from the relaxation of structural commensurability constraints at constituent heterobilayer interfaces. This is crucial because the multilayer stacks are experimentally realizable through layer-by-layer transfer of component materials that have already been deposited into a heterostructure stack. Interfaces without barriers were created in an atomic simulation environment (ASE) [38] by combining orthogonal fcc (111) and bcc (110) slabs with vacuum layer of height 25 Å inserted along the *c*-axis in each case. After inserting monolayer hBN between two orthogonally stacked dissimilar surfaces, the resulting trilayer interface is made from different materials components and coupled together by van der Waals interactions. These stacks can yield the FM [33,36,37] or the AFM [39] ground state; however, a reversible current-induced switch between the AFM and FM states is also a unique possibility, as recently found with CrI_3_ [40].

Collinear magnetization calculation was first performed without spin orbit coupling (SOC) for the structure relaxation. The optimized interlayer distances and atomic coordinates were obtained using the nonempirical spin density form of the van der Waals density (svdW-DF2) [41] using the PWSCF code of the QUANTUM ESPRESSO suite [42,43]. Electron–ion interactions were described using PAW potentials [44]. Cut off limits of 70 and 800 eV for the kinetic energy and charge density expansions were sufficient to converge the electronic energy and Hellman–Feynman forces to within 10^−12^ eV and 10^−3^ eV/Å. The Brillouin zones were sampled with a 12 × 12 × 1 Monkhorst–Pack *k*-point grid [45]. Electron states were treated as spinors with a double group symmetry and were populated using Marzari–Vanderbilt cold smearing at a width of 14.7 mRy [46]. The relaxed structures were then used as the input charge density for the second calculation of the simulated structures in the non-collinear magnetic ground state.

In the noncollinear magnetization calculation, the effect of SOC may be included via relativistic pseudopotentials. SOC increases Kramers degeneracies through the splitting of electron states that degenerate when SOC is not included. The noncollinear magnetization calculations were performed without including van der Waals interaction, with all other DFT tuning parameters unchanged. Additional structure relaxation effects due to the inclusion of SOC were ignored. With the spin quantization direction fixed to the *z*-axis of the stack, the atom-resolved magnetic moments at the DFT ground state were used as the input for the classical dynamics of the atomistic spin Hamiltonian. In addition, the total magnetization for each structure was resolved to the magnetic moment per atomic site. This forms the basis of the obtained DFT-level properties that need to be retained, which then determines the most appropriate coarse-grained approach. As equally demonstrated by Hadley et al. [47] in their simulations of water, the loss of details in the coarse graining does not allow for all physical properties to be matched to those of fine-grained models. This is because some of the behaviors of the spin system are mutually exclusive in relation to parameter fitting. This is circumvented here since our objective is to study the spin textures on a 2D lattice. Hence, the most appropriate method corresponds to the fitting of the spin Hamiltonian to the total magnetization of the fine-grained DFT model. The absolute magnetic moments of the DFT ground state are used to construct the Hamiltonian used for investigating the classical spin dynamics considered herein.

### 2.2. Skyrmion Formation in the Spin Hall Regime

The approach of Evans et al. [48] for analyzing the local intrinsic fields in magnetic systems was adopted to define a set of interaction parameters for studying the dynamic evolution of localized spins. By analogy to chemical models, the interaction parameters behave as an effective “force field”. Our “force field” is therefore the spin-space magnetic Hamiltonian *H*, which is obtained as the sum of the exchange energy (*H_exch_*), the anisotropic energy (*H_anis_*), and the applied external magnetic field (*H_ext_*). Nonmagnetic (NM) interaction contributions from the Coulomb term are ignored such that the magnetic Hamiltonian is given in spin space as follows:

(1) $$H=H_{exch}+H_{anis}+H_{ext}$$

The major contribution to the spin Hamiltonian is the exchange energy, and it is dominated by the Heisenberg exchange energy. The Heisenberg exchange energy was obtained from DFT calculations of the non-collinear ground state, and it originates from the symmetry of the electronic wave function and the Pauli exclusion principle. The exchange energy, *H_exch_*, of two interacting nearest-neighbor spin moments, **S***_i_* and **S***_j_*, whose interaction strength is *J_ij_*, is expressed as follows:

(2) $$H_{exch}=-\frac{1}{2}\underset{i\neq j}{\sum }J_{ij}S_{i}\cdot S_{j}$$

In FM materials, where spins align parallel to one another and to the external magnetic field, *J_ij_* > 0, whereas in AFM materials, where spins align antiparallel to one another, *J_ij_* < 0. Usually, there is a strong dependence of *J_ij_* on the distance separating two interacting spins. As such, a realistic computation of pairwise interactions is expensive, since spin interactions extend over several atomic spaces, leading to the inclusion of further pairwise interactions.

The summation in Equation (2) considers near-neighbor spins only. As such, the exchange energy of two near-neighbor spins at sites *i* and *j* depends only on their relative orientation and not their direction. In the noncollinear magnetic ground state, where no spin texture exists, the Heisenberg exchange is isotropic. This allows the strength of the exchange interaction to be treated as in magnetic materials and expressed as a symmetric rank two tensor.

(3) $$J_{ij}^{S}=\left[\begin{array}{ccc} J_{xx} & J_{xy} & J_{xz}\\ J_{yx} & J_{yy} & J_{yz}\\ J_{zx} & J_{zy} & J_{zz} \end{array}\right]$$

where this allows for tensorial description of asymmetric exchange $J_{ij}^{A}$:

(4) $$J_{ij}^{A}=\left[-\begin{array}{ccc} 0 & D_{ij}^{z} & -D_{ij}^{y}\\ D_{ij}^{z} & 0 & D_{ij}^{x}\\ D_{ij}^{y} & D-{\;}_{ij}^{x} & 0 \end{array}\right]$$

For *z*-axis spin quantization, the exchange energy is obtained from DFT calculations as follows:

(5) $$H_{exch}^{DM}=D_{ij}\cdot \left(S_{i}\times S_{j}\right)$$

where $D_{ij}=D^{k}\cdot \left(\hat{z}\times u_{ij}\right)$. Furthermore, $D^{k}$ and$\;\hat{z}\times u_{ij}$ denote the strength and unit vector of the Dzyaloshinskii–Moriya interaction between spins located at sites *i* and *j*. The external magnetic field H contributes a finite magnetic field energy, $H_{ext}$, to the spin Hamiltonian, which is denoted as follows:

(6) $$H_{ext}=\mu_{s}\underset{i}{\sum }S_{i}^{z}\cdot H$$

Since there is no intrinsic (or global) crystal structure in artificially stacked Fe/hBN/HM heterostructure multilayers, the contribution of anisotropy energy to the spin Hamiltonian for preferential spin alignment to the spin quantization direction (i.e., *z*-axis of the stack) was obtained in terms of the uniaxial single-ion anisotropy as follows:

(7) $$H_{anis}=-K_{u}\underset{i}{\sum }{\left(S_{i}\cdot \hat{z}\right)}^{2}\;$$

The usual starting point in atomistic modeling of the motion of coupled (or uncoupled) and damped (or undamped) spin magnetic moments is the Landau-Lifshitz (LL) equation [49],

(8) $$\frac{dM}{dt}=-\gamma \left[M\times H_{eff}\right]-\gamma \frac{\lambda }{M_{s}}\left[M\times \left(M\times H_{eff}\right)\right])$$

where M is the magnetization, $M_{s}$ is the saturation magnetization, $H_{eff}\;$is the effective magnetic field, *λ* denotes a phenomenological damping parameter, $\gamma =g\left|e\right|/2m_{e}$ is the gyromagnetic ratio (where the Landé *g*-factor for free electrons is 2), and *e* denotes the charge of the electron of mass *m_e_*. In order to investigate the time evolution of the normalized magnetization m from the injection of polarized spin current density (***J_s_***) into the HM layer due to a charge current density (***J_c_***) flowing through the Fe(110) layer akin to the SHE, we first considered the Landau–Lifshitz–Gilbert (LLG) equation [50].

(9) $$\frac{dm}{dt}=-\frac{\gamma }{\left(1+\alpha^{2}\right)}\left[m\times H_{eff}\right]-\frac{\gamma }{\left(1+\alpha^{2}\right)}\alpha \left[m\times \left(m\times H_{eff}\right)\right]\;,$$

where **m** = **M**/M_s_. By contrast, the spin dynamics in FM/HM/FM heterostructures under FM resonance is described by incorporating the additional term *τ* in Equation (9) to capture the effect of spin orbit torque (SOT) in terms of switching magnetization in the FM free layer. With the gyromagnetic ratio γ replaced with γ/(1 + α^2^) and setting *α* = *λ* (α is the dimensionless Gilbert damping constant), this makes the spin dynamics tractable when using the Landau–Lifshitz–Gilbert–Slonczewski (LLGS) equation:

(10) $$\frac{dm}{dt}=-\gamma \left(m\times H_{eff}\right)+\alpha \left(m\times \frac{dm}{dt}\right)+\frac{\gamma }{\mu_{0}M_{s}}\tau$$

where the Slonczewski torque τ is partitioned into adiabatic and non-adiabatic torques as follows:

(11) τ=a(j)[m×(m×p)]+b(j)(m×p)

where p denotes the spin polarization in the Fe(110) layer, while a(j) and b(j) are current-dependent functions for the parallel (i.e., in-plane) and perpendicular (i.e., out-of-plane) torques.

### 2.3. Spin Memory Loss at Interfaces

In a spin Hall regime, the flow of an injected spin current causes a net transfer of angular momentum at interfacial regions between FM/hBN and hBN/HM interfaces of the stack and STT vanishes (*τ* = 0). Thus, the LLGS model of the dynamic evolution of atomistic spins treats the ground state of the frustrated ferromagnetic stacks as a spin ice model. As such, the spin dynamics are simulated using an effective magnetic field **H*_eff_*** at zero STT. Nevertheless, the effect of SOC in the HM layer causes electrons with different spin properties to deflect in different directions in a SHE regime. This causes a pure spin current to be generated in the NM material that is oriented transverse to the applied charge current. Pure spin currents can carry information with minimum power dissipation and can also manipulate the magnetization of an adjacent FM layer. Spin precession in a FM layer can transfer angular momentum to conduction electrons in an adjacent NM layer when of a HM species. Thus, van der Waals interaction plays no role in determining the dynamic transition from a soft to hard magnetic state. The spin current density, **J_s_**, injected into the HM layer is given as follows:

(12) $$J_{s}=\frac{ћ}{4ᴨ}\left(g^{↑↓}\hat{m}\times \frac{d}{dt}\hat{m}\right)$$

where $\hat{m}$ denotes the unit vector of the magnetization and g^(↑↓) is the spin mixing conductance [51]; however, the efficiency of spin transport across HM/FM and hBN/HM interfaces is determined by the effective spin mixing conductance (g_eff^(↑↓)) [51]. In a spin Hall regime, the heterostructure contains only one FM/HM interface. The first term in Equation (9) accounts for band structure mismatching and spin decoherence in HM/hBN and hBN/Fe(110) heterobilayer interfaces and interfacial effects for polarization of the FM layer. The second term, which accounts for the spin accumulation at constituent interfaces, is also sensitive to the transparency of the interface (see Section 3.4).

Table 1 shows the input parameters used for the LLGS simulations in Spirit [52]. The initial spin moment at ground state, Rashba SOC parameter (αR), bulk anisotropy of the FM layer, and magnetic interaction parameters for the symmetric (i.e., *J_ij_*) and asymmetric (i.e., *D_ij_*) exchange fields were derived from DFT. Using the site-resolved magnetic moments at the ground state that were derived from non-collinear magnetic calculations as the starting spin configuration in the FM-state, we implemented numerical solutions of the equilibrium spin system as stochastic solutions of the LLGS equation over a duration of 150 ps. Time evolution of the spin system was computed using parameters of the SHE in the presence of a topological skyrmion. To describe the effect of the spin transfer torque (STT) in terms of the switching of magnetization, an additional term was added to the torque field. The added torque is denoted by τ in Equation (3). It represents the current-induced STT for the FM layer. The Slonczewski spin torque was considered by adding a new field to the effective field. Magnetic hysteresis loops and coercivity were calculated and the dynamic stability of isolated skyrmions was obtained by studying the delicate balance between the interactions of *J_ij_* and *D_ij_*, the Zeeman field, and the external magnetic field.

Within the Hall effect regime, there is a net flow of angular momentum at the interfacial regions between the FM/hBN and hBN/HM interfaces of the stack. Thus, it is crucial to note that the direction of deflected electrons is normal to the applied uniform electric field ($E=E_{0}\hat{x}$) relative to the spin quantization direction ($p=p_{0}\hat{z}$). The spin current flows perpendicular to the in-plane direction of the interface. This ensures that spins aligned parallel to the *z*-axis of the interface correspond to the collinear magnetism in the stack [58]. This allows insights regarding the spectroscopic signatures of the spin current generated in the HM layer to be obtained. The excitation of magnetization dynamics by the SOT in the adjacent FM layer is obtained by calculating the interfacial spin memory loss (SML) during SHE. At FM/hBN/HM interfaces, the SML (denoted by ζ) is obtained as the effective reduction in the magnitude of the spin polarization (**p**) using the following definition:

(13) $$\zeta =\frac{\left|p_{\mathrm{NM}}^{\mathrm{out}}\right|}{\left|p_{\mathrm{FM}}^{\mathrm{in}}\right|}=\frac{\left|p_{\mathrm{NM}}^{\mathrm{out}}\left(d_{\mathrm{HM}}=1\;\mathrm{ML}\right)\right|}{\left|p_{\mathrm{FM}}^{\mathrm{in}}\right|}\;$$

where $\left|p_{\mathrm{FM}}^{\mathrm{in}}\right|\;$denotes the polarization generated by an infinitely thick FM layer, and $p_{\mathrm{NM}}^{\mathrm{out}}\left(d_{\mathrm{HM}}=1\;\mathrm{ML}\right)$ denotes the polarization of the current in the NM lead after the insertion of 1 ML of the HM as given by Dolui and Nikolic [59]. For FM layers of a finite thickness, the limit $\left|p_{\mathrm{FM}}^{\mathrm{in}}\right|=\left|p_{\mathrm{NM}}^{\mathrm{out}}\left(d_{\mathrm{HM}}=0\;\mathrm{ML}\right)\right|$ implies that ζ = 1 for an infinite Fe slab and correlates with ζ in the ISHE. Thus, ζ denotes the amount of spin current that has been absorbed at the interface and the upper bound of the SML, while ζ characterizes the spin injection efficiency of a given interface. The difference 1−ζ gives the interface spin transparency T*_int_*, which estimates the spin Hall efficiency of the interface. Intrinsic coupling exists between the electronic spin and orbital degrees of freedom in the bulk and surface geometries of HM layer species. As such, interface-sensitive phenomena manifest in the spin transport properties derived from over-layer physisorption of the FM layer on a HM substrate (i.e., HM/FM bilayer) or two dissimilar NM layers (e.g., NM/HM bilayer). This has been analyzed to unravel the contributions to the spin Hall conductivity (SHC) [60]. Since the transverse Hall voltage generated by the longitudinal charge current is sensitive to the net magnetization (or spin polarization) created by SOC-induced splitting of the transition metal *d*-band center at the interface, more detailed electronic structure, magnetic properties, and SOC-induced spin textures may be explored to obtain deeper insights. With the spin quantization axis fixed to the *z*-axis of structures, variations in ζ as the interfacial structure changes from HM/FM and NM/HM due to monolayer hBN incorporation may be used to determine SHA. Interfacial spin transparency T*_int_* may be analyzed to yield deeper insights regarding the AHE and SHE for electrons crossing FM/HM and NM/NM interfaces.

## 3. Results and Discussion

### 3.1. Distribution of Magnetic Moments at Ground State

The distribution of local magnetic moments shows that both the total (M_T_) and absolute (M_A_) magnetization densities favor collinear magnetization at a ground state. This yields a saturation magnetization where **M_T_** = (0, 0, −M_s_) in each stack, indicating the spin-flip FM ground state. It is found here that due to the large *D_ij_*, the spin-flipped ground state favors the dynamic formation of a MS. The magnetic properties of HM-based stacks are understood here from two perspectives. Firstly, from the view of the electron dynamics, we consider the electric and magnetic fields that emerge from the modification of spin orientation as induced by the Lorentz force relative to the spin quantization direction [61,62]. In this viewpoint, we consider that the flipped-spin ground state suggests that the applied magnetic field deflects electrons and that this effect is the skyrmion SHE [63]. Secondly, a finite voltage drop is expected to develop along the direction perpendicular to the direction of flow of spin current, which is measurable as the transverse Hall voltage.

Besides, the smoothness of the skyrmion lattice suggests that any adjustments to the spins of electrons to the magnetization of the MS state must occur via an adiabatic process. The force resulting from the transfer of the spin angular momentum from conduction electrons to localized atomic magnetic moments causes localized spin texture domains to move. In the absence of the MS phase, the effect of this resultant force is responsible for the spin backflow phenomenon reported recently for the tunneling spin current in the related FM multilayer stacks [36]. It has been found that M_A_ is sensitive to the HM species, while their direction shows uniform alignment. The results show values of 37.86 μ_B_/cell (Ta-capped), 29.98 μ_B_/cell (Pd-capped), and 32.04 μ_B_/cell (Pt-capped) at the ground state. These results suggest that the application of an external magnetic field will enhance the total magnetization by aligning local spin moments along the z-direction, since the ground state is FM. By contrast, the total magnetization is non-uniform. We obtained the reversal of the magnetization direction relative to the spin quantization axis as a unique feature of the frustrated FM stacks. The magnitude of the total magnetization exhibited strong sensitivity to the chemical identity of the atomic species of the HM capping layer. For instance, total magnetization values of −21.36 μ_B_/cell (Ta-capped) and −9.88 μ_B_/cell (Pd-capped) were obtained. By contrast, the total magnetization of −10.89 μ_B_/cell in the Pt-capped stack is nearly of equal magnitude to the magnetization in the Pd-capped stack. To understand the similarity between the total magnetization in the Pt- and Pd-capped stacks, it is crucial to first understand the effect of SOC on the electronic structures of both Pt and Pd. SOC is strongest in heavy elements, where electrons acquire large velocities near the nucleus. Thus, insights into the effect of SOC may be obtained by studying spin-resolved electronic band structures.

**Figure 1:**
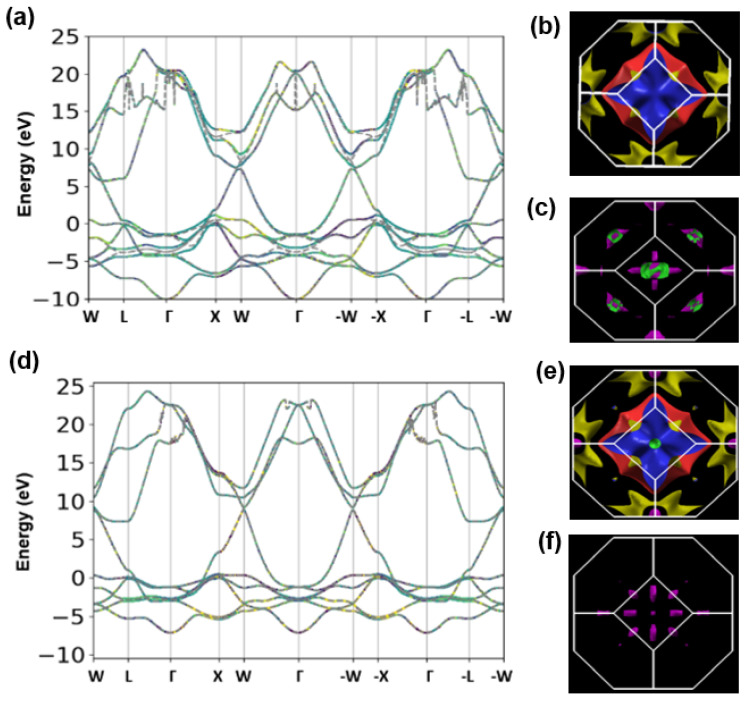
The electronic band structures (**a**,**d**), Fermi surfaces (**b**,**e**), and spin Berry phase isosurface maps (**c**,**f**) for Pt (top panels) and Pd (bottom panels).

Figure 1 shows the electronic structures and the signatures of spin transport for Pt (top panels) and Pd (bottom panels). Figure 1a,d show the band structures with and without the inclusion of SOC for Pt and Pd. In addition to the energy eigenvalues, the spin character is also displayed in color in the band structure plots. Red denotes spin upward (↑↑) states and blue denotes spin downward (↓↓) states. The band structure without SOC is shown as dashed grey lines for paired spin (↑↓) states. Although SOC can increase Kramers degeneracies between electron states that are protected by time reversal symmetry when it is absent, the SOC had no effect at the Γ-point for Pt and Pd. The band structures Figure 1a,d in show band points as superpositions of red, blue, and grey lines under an imposed time reversal symmetry constraint where *E*(*k*) = *E*(−*k*). In Pt (see Figure 1a), there is a band crossing at a W-point approximately 8.0 eV above the Fermi level; however, the degeneracy is lifted by the SOC and the bands become split by 1 eV at this point.

Crucially, we also find that without SOC, two of the 5*d* band states in Pt degenerate along the entire Γ−X line. The degeneracy is lifted when SOC is included. In Pd, by contrast, this effect is also present, although it is vanishingly small at the W-point, while all the 5*d* band states along the entire Γ−X line are degenerative and insensitive to SOC. This shows that, in the absence of SOC, the electron states in HM species are not unique eigenstates of the spin projection operator. This is because for a spin quantization axis fixed along the *z*-axis, electron eigenstates for the ↑↑, ↓↓, and ↑↓ spin couplings are invariant. The invariance is ascribable to the preservation of the spatial inversion that is symmetric in the local structures of Pt and Pd. This trend is also due to the inability of SOC alone to break time reversal symmetry in bulk HM species, even though it causes Zeeman spin splitting.

The 5*d* bands of both metals straddle the Fermi level around the X and L points of the Brillouin zone and these bands bridge the conduction and valence bands. These electronic signatures are important consequences of the strongly correlated nature of electron states in the HM species. We show in Section 3.2 that different interfaces yield different spin responses, which thus influences the interfacial spin transparency (T*_int_*). We attribute these effects to the SOC-induced spin splitting of *d*-band states, which underpins the perpendicular M_A_ and M_T_ values observed in heterobilayer interfaces. The Fermi surface (Figure 1b,e) and the Berry phase curvature (Figure 1c,f) are shown as projections on a truncated octahedron representation of the Brillouin zone. Their isosurface distributions are similar. Both reflect the simultaneous presence of spatial regions of extended localization of electron states and regions of disconnectedness. These reflect the nearly-free character of the Fermi surface in Pt and Pd. The dependence of the low-energy dispersion on HM species shows that SOC has no effect on the ground state. Because of the inability of intrinsic SOC to lift spin degeneracies as an exclusive effect, other relevant spin signatures that are detectable in spin magnetotransport experiments are also explored below.

**Figure 2:**
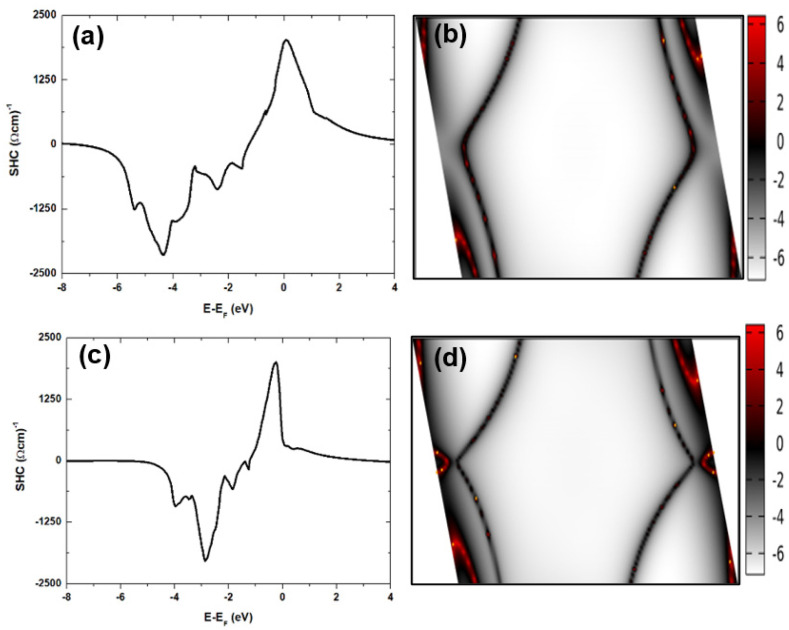
Signatures of spin transmission in Pt (top panels) and Pd (bottom panels), showing the spin Hall conductivity (**a**,**c**) and reciprocal space contour maps of the spin texture (**b**,**d**).

Figure 2 shows the signatures of spin transmission in Pt and Pd in terms of the spin Hall conductivity and the spin texture as a projection of the reciprocal space contour map. Figure 2a,c show the calculated spin Hall conductivity (SHC) for Pt and Pd at 0 K. At the Fermi level, our calculations show values of 1977 Ω^−1^cm^−1^ for Pt and 449 Ω^−1^cm^−1^ for Pd. Our results are consistent with the prior values of 2000 Ω^−1^cm^−1^ [64] and 2170 Ω^−1^cm^−1^ [65] for Pt and 330 Ω^−1^cm^−1^ [66] for Pd. By contrast, the room temperature measurement of 2.4 × 10^4^ Ω^−1^cm^−1^ for Pt is far higher than the calculated value of SHC at 0 K [67]. We note that the peak positions in the SHC plots shown in Figure 2a,c are shifted above (or below) the Fermi level in Pt (or Pd). Considering the minimalistic differences between the Pt and Pd band structures (see Figure 1a,d), our observations of a small SHC signal at the Fermi level are attributable to the differences in their spin textures.

Figure 2b,d show the spin textures in fcc bulk forms of Pt and Pd. The spin texture is inverted disproportionately between the valence and conduction band states. Red and white denote positive and negative spin eigenstates of S_z_. These show disproportionate inversion of S_z_ insofar as positive S_z_ denotes spin states localized at special Brillouin (BZ) points along the edges of the hexagonal reciprocal lattice. Planes that define the edges of the hexagonal reciprocal lattice are characterized by zero S_z_, with negative spin eigenstates localized mainly at the center of the distorted hexagon. Thus, although Pt and Pd are intrinsically NM species, there are subtle differences in their spin textures, which further underscores the role of SOC in their carrier transport. The observation of a non-zero spin texture in the HM species is important for the emergence of skyrmions in magnetic stack systems. Direct observation of such spin textures could facilitate the advancement of spintronic technologies [68], such as magnetic data encoding to spin domains. Considering that the electronic ground state in bulk Pt is just like that in bulk Pd, both metals satisfy the Stoner criterion for weak ferromagnetism due to the SOC-induced splitting of the degenerate 5*d* bands. Figure 1a,d show the 5*d* bands as localized states that are deep into valence band and up to 5 eV below the Fermi level. Since both metals are at the verge of ferromagnetism, the large static (and Pauli) susceptibility observed in Pt and Pd is attributable to Stoner enhancement of magnetic susceptibility [69]. Figure 2 shows the electronic structure and spin transport signatures in bulk Pd for comparison with the electronic structure of Pt. Clearly, bands located below the Fermi level are closely spaced “flat” bands. Such bands are expected to result in large densities for states near the Fermi level, which is in line with the Stoner criterion for ferromagnetism. The similarities between Pt and Pd ground states mean that heterostructures that contain HM species form heterobilayer interfaces with Fe and hBN and retain the FM ground state. Strong intrinsic SOC interaction in HM species causes the spontaneous splitting of bands. When combined with broken inversion symmetry at constituent heterobilayer interfaces, the multilayer stack platform is suitable for the emergence of intriguing spin phenomena. These interfacial spin phenomena are explored below, and the Pt layer of the stack is found to support nonvanishing net magnetization for a skyrmion affected by the SHE, even though it is an intrinsically nonmagnetic component of the stack.

### 3.2. Interface Spin Transparency

In a recent review, Jiang et al. [70] have detailed the current understanding of skyrmions in magnetic multilayers. Similarly, Kim [71] also reviewed skyrmions and Hall transport. These studies have underscored the importance of parity violations of the electron state at the interfaces in the magnetic phenomena of coupled nanomaterials. For instance, the antisymmetric exchange interaction (i.e., DMI) stabilizes chiral spin textures in multilayer systems. In Ni/Co/Pd/W(110) multilayers, chemisorption of oxygen leads to a large DMI [72]. In addition, the symmetric part of an anisotropic DMI is an important ingredient for stabilizing topologically nontrivial magnetic textures in thin films [73], but the DMI must combine with symmetric Heisenberg exchange to stabilize a skyrmion in a multilayer [74]. These ingredients constitute the crucial internal fields required for a skyrmion to form. Thus, insights to the skyrmion Hall transport in Fe(110)/hBN/HM stacks may be developed from considerations of the internal field effects of the broken structural inversion and time reversal symmetries at constituent heterobilayer interfaces.

**Figure 3:**
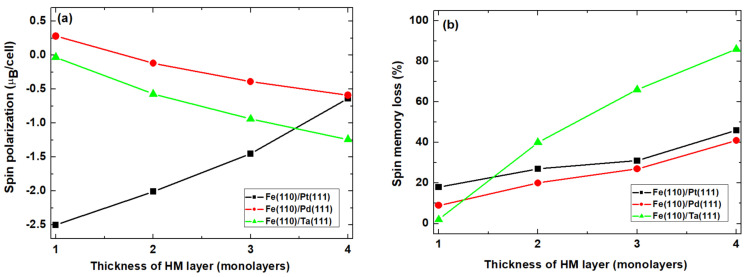
Heavy metal layer thickness dependence of the absolute spin polarization (**a**) and spin memory loss (**b**) in barrierless heterobilayer interfaces. The points plotted on the axis of increasing spin polarization correspond to increasing thicknesses of HM species in monolayers.

Figure 3 shows the interfacial signatures of spin transport across barrierless heterobilayer interfaces and their dependence on the chemical species of the HM layer. The absolute magnitude of the spin polarization (**p**) (Figure 3a) and spin memory loss (ζ) (Figure 3b) show strong dependence on the thickness, as well as the chemical species of the HM capping layer of the interface. Interfacial **p** and ζ increase in magnitude with increasing thickness in the Pt-capped interface, whereas **p** decreases with increased Pd and Ta thicknesses. By contrast, the general trend line behavior of ζ shows a proportionate increase with an increasing thickness of each HM layer type (Figure 3b). Nevertheless, as the thickness of the capping layer increases, the Ta-capped interface exhibits a substantially rapid increase in ζ relative to the Pt and Pd capping species. At a 4-ML thickness, the Pt- and Pd-capped interfaces have the same magnitude for interface spin polarization (**p**). These observations are understandable in terms of the differences between the electronic structures of bulk and surface structures in terms of making up the interface, as the layer thickness is increased. The opposite trends in the magnitudes of the interface spin polarization in Pd- and Pt capped interfaces at thicknesses of 1 and 3 ML can be understood by noting that surface electronic effects are expected to be dominant at low thicknesses. On the contrary, bulk electronic effects are expected to dominate at 4 ML. Hence, the magnitude of **p** is invariant for Pd and Pt at 4 ML (see Figure 3a), as would be expected from the electronic structures of the two fcc bulk metals.

Properties such as interface quality, interface spin transparency, and the electrical resistance of the interfaces determine robustness in terms of device performance and microelectronic reliability in spintronic applications. The band structure mismatch at the interface between HM species and monolayer hBN, i.e., a 2D semiconductor, yields resistive contacts due to formation of large Schottky barriers. Monolayer hBN is known to play a nontrivial role in the generation of coherent spin tunneling [37]. This is because it minimizes resonance tunneling effects [75]. It is still important to unravel the interfacial effects on the emergent magnetic properties. In the Pd- and Ta-capped barrierless interfaces, Figure 3 reveals significantly attenuated spin polarization. This is also accompanied with an increase in ζ as the thickness of the HM layer increases (see Figure 3a). We attribute the attenuated spin polarization density to spin canting. This effect, which can originate from the dissipation of spin angular momentum due to magnetic exchange interactions at heterobilayer interfaces and interfacial band hybridization [76], perpendicular magnetic anisotropy PMA [77], determine spin filtering and transmission efficiency. Quantum effects like spin accumulation and spin backflow also affect ζ and T_int_. At finite temperatures, microscopic processes such as field-induced spin flip scattering [78] and magnon scattering [79] dominate spin canting.

**Figure 4:**
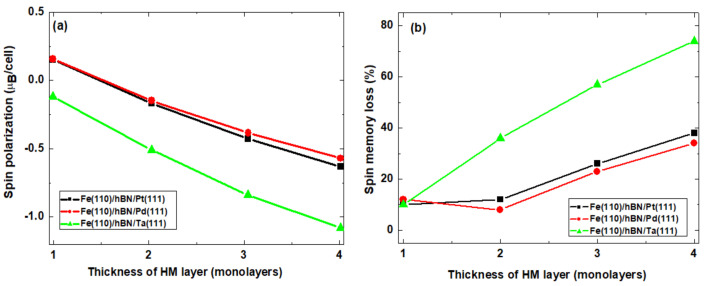
Effect of the monolayer hBN barrier layer on the thickness dependence of interfacial spin polarization (**a**) and spin memory loss (**b**) for different heavy metals in capped heterobilayer interfaces.

Recent studies have shown that spin phenomena at an interface are affected by interface alloying [80,81,82]. We have also unraveled the effects of monolayer hBN insertion into heterobilayer interfaces as a realizable variant of interface alloying. Figure 4 shows the thickness dependence of interfacial **p** and ζ for different heavy metals capped in heterobilayer interfaces in the presence of the monolayer hBN barrier layer. After the insertion of the hBN monolayer into the Pt-capped ferromagnet, a significant change in **p** is observed. This manifests as a continuous decrease in the magnitude of **p** as the thickness of the Pt layer increases. By contrast, when the monolayer hBN barrier is inserted into either the Pd- or Ta-capped interface, the magnitude of p increases relative to the corresponding response in barrier-less interface (see Figure 4a). Thus, although the trend line dependence of **p** on the thickness of the Pd and Ta cap is unchanged, the magnitude of **p** increases by a factor of ~3 for both Pd and Ta. Figure 4b shows the corresponding effect of monolayer hBN on ζ. To understand the role of the hBN layer in a Fe(110)/hBN/Pt(111) interface, a comparison of Figure 2 and Figure 3 shows simultaneous decrease (increase) in **p** (ζ) as the thicknesses of the HM layer increases. Crucially, the interfacial spin transparency ζ shows no sensitivity to the chemical species of the HM layer at 1 ML. These show that the hBN monolayer has a significant effect on the spin signatures at the interface and determines the overall spin transmission efficiency of the interfaces.

### 3.3. Skyrmion Induced Transition from Soft to Hard Ferromagnetic State

**Figure 5:**
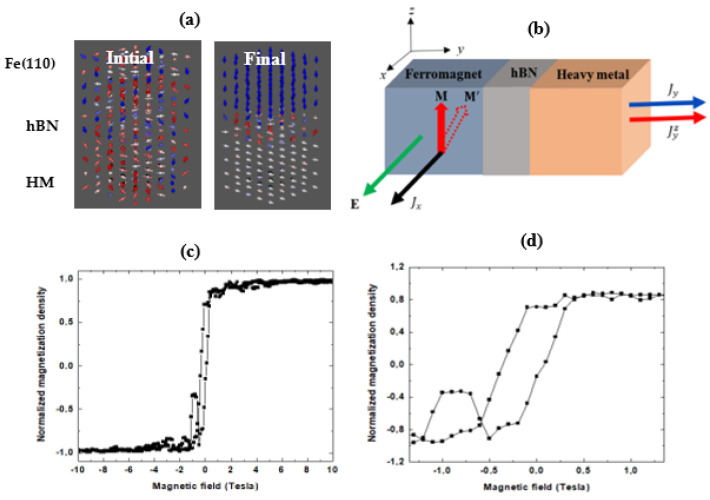
Ising-like spin ice model of the magnetic state in Fe(110)/hBN/HM stacks obtained from layer-resolved spin orientations at ground state under the spin Hall regime. Red denotes spinning upwards (↑↑) and blue denotes spinning downward (↓↓) (**a**) for schematics of the initial (i.e., unfiltered) and final (i.e., filtered) spin configurations in the measurement geometry of the spin Hall effect (**b**) and the magnetic hysteresis loop (**c**). A zoomed plot of the hysteresis loop around **H**,**M** ≡ 0,0 is shown (**d**).

Figure 5 shows the dynamic properties of Fe(110)/hBN/*HM* magnets in terms of the SHE measurement geometry. Figure 5a shows an Ising-like spin ice model of the magnetic state in the Fe(110)/hBN/HM(Pt) stack obtained from layer-resolved spin orientations at a ground state in the spin Hall regime. It is clear from Figure 5a that no long- or short-range order exists in the local arrangement of atomic spins of the initial configuration. The fact that the net magnetization is non-zero in the Heisenberg spin model of the Fe(110)/hBN/HM(Pt) stack at the ground state, despite the disordered spin geometry, suggests the presence of some residual spin order. The final spin configurations in specific atomic layers of the Fe(110)/hBN/HM stack are ordered due to the different directions of the flow of the charge current and injected spin current. In the SHE measurement geometry shown schematically in Figure 5b, **M** denotes the magnetization vector corresponding to spin polarization ($p=p_{0}\hat{z}$) due to spin quantization along the *z*-axis of the stack. The directions of the applied charge current density (**J_c_**) and external magnetic field (**H**) for the calculation of the hysteresis loops are reversible; however, the magnetization **M** is collinear with field **H**. The HM species are nonmagnetic. In addition, the wide band gap of the hBN layer endows it with high resistivity. Thus, the apparent intrinsic order in directions of spin is determined by the direction of flow of spin angular momentum as the spin current is injected in the HM layer.

Figure 5c shows the magnetic hysteresis of the disordered spins geometry in the spin Hall regime. The hysteresis loop is shown as a zoomed plot around (H,M ≡ 0,0) in Figure 5d. The loop is not symmetrical about the M and H axes. The following unique features are observable: The magnetization density saturates at a relatively low applied magnetic field of ~2 T. The estimated magnetic coercivity of 0.5 T is low, while the calculated retentivity (i.e., remanence magnetization is 72% of the saturation magnetization. In reverse magnetic fields, magnetization reaches maximum saturation at ~1.0 T. These scenarios suggest that the stack is easy to magnetize and demagnetize. As a result, any associated domain wall motion is easy. Thirdly, the hysteresis loop is qualitatively different from the skyrmion with the Hall regime. The low field magnetic hysteresis shown in Figure 5d is significantly different from the characteristic behavior of weak ferromagnets.

Fluctuations in the low-field magnetization density reach a peak between −1.1 and −0.5 T, although constant magnetic susceptibility emerges at an external field of −0.75 T. The constant magnetic susceptibility persists up to −1.0 T. This characteristic feature is only observed under an antiparallel alignment of the magnetic field. Its absence under parallel alignment of the magnetic field suggests that it is a dynamic effect at the magnetic state, which is insensitive to the magnetic history. The magnetization density of this dynamically created magnetic state for both parallel and antiparallel spin orientations reveal FM moments with a spin flip transition at H_sf_ = ~0.5 T. The weak ferromagnetism manifests as spontaneous magnetization and magnetic hysteresis near the zero field strength (Figure 5d) and is driven by the interplay between the easy axis magnetization in the Fe(110) layer and the antisymmetric exchange interactions at heterobilayer interfaces. This metamagnetic transition leads to formation of dynamic magnetic behavior. Finally, we demonstrate that the dynamic magnetic behavior leads to formation of the MS state and switches the weak ferromagnetism from a soft to hard magnetic state.

**Figure 6:**
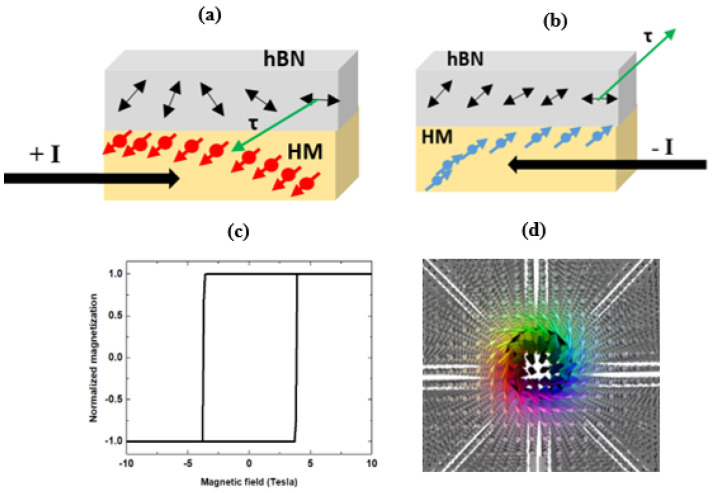
Directions of the SOT for positive (**a**) and negative (**b**) spin current injection through the hBN layer into the HM layer in the spin Hall regime, the M-H loop of the current-induced hard magnet (**c**), and a dynamically stable magnetic swirl at 25 T with spherical texture of a spiral MS (**d**).

Figure 6a,b display the direction of the injected spin current that leads to formation of an energetically unstable chiral MS. The hysteresis loop obtained in the presence of the MS denotes a hard magnet state (see Figure 6c). Injection of positive (or negative) pulse current of yields invariant hysteresis loops. Clearly, the magnetization density vanishes at ~3.87 T in the positive (+I) drive current. In the regime of negative (−I) drive current, the m drops to zero at −3.7 T. The local structure of the MS is shown in Figure 6d. In addition, the magnetic retentivity is 100% with a coercivity of 3.7 T. Relative to the coercivity of ~0.5 T of the soft magnetic state, 3.7 T is high and suggests the formation of a hard magnet, which, once magnetized, is not easy to demagnetize. The calculated coercivity of 3.7 T corresponds to a non-zero residual magnetic flux. This is the magnetic flux that remains at the final spin configuration (see Figure 5a) when **m** = **p** = 0; however, when **m** = **p** ≠ 0, the injection of spin current is 100%. The above results characterize the exchange bias as a dynamic shift in the hysteresis loops of the ferromagnetic stack. This shift arises from the interfacial exchange coupling between adjacent ferromagnetic and antiferromagnetic layers, and such shifts are an integral to spintronic devices.

We now show that the nonmagnetic character of the hBN layer, which causes the randomized spins in the interfacial between Fe and Pt in the final spin configuration (see Figure 5a), induces dynamic responses. Following the analysis of the quantum anomalous Hall insulating state by Pan et al. [83], a similar argument is adopted here to keep the induced dynamic responses of spins tractable from the computed hysteresis loops. This allows the spin transport phase in Fe(110)/hBN/HM ferromagnets to be represented on the 2D lattice in order to capture the formation of the MS transport state. This 2D lattice representation of the spin transport phase is desirable for realizing the collective excitation associated with the field response. Figure 6d shows a 2D lattice representation of the spin transport phase in the hard magnetic state. Owing to the interplay between the magnetic exchange bias and strong interfacial DMI, the spin texture of the transport phase yields the skyrmion. We now elucidate deeper insights to the dynamic response of the frustrated van der Waals ferromagnets to the injected spin current density. This is derived by considering that the soft FM state (Figure 5c) is attributed to the disordered spins and to the effect of geometric frustration on the resultant magnetization [84]. These combined effects signal the emergence of the nontrivial dynamic magnetic state. Importantly, the magnitude of current-induced magnetization is unchanged after reversing the direction of the injected spin current. This leaves the hysteresis loop and magnetic properties invariant under current reversal, suggesting that the exchange interaction effect is insensitive to the direction of current flow ferromagnets. Instead, it depends on the ordering of atomic spins relative to the direction of the magnetic field. Thus, it is useful to compare the above findings with results of recent experiments with somewhat similar van der Waals ferromagnets.

The experimental magnetic hysteresis loop measured in the FM alloy CoFeB/IrMn/Pt trilayer stack exhibits a strong exchange bias. The strong bias is due to the injection of a polarized spin current into the AFM layer and the resulting SOT on magnetic moments [85]. Thus, further insights into the current-induced switching of magnetic properties in capped HM stacks from a soft magnetic state (Figure 5c) to hard magnetic state (Figure 6c) is obtainable by first noting the strong magnetic exchange bias that is exclusively due to the presence of the skyrmion. Despite the injection of a polarized spin current into the HM layer through the hBN monolayer, the origin of the strong exchange bias cannot be attributed to the associated STT of the injected current. This consists of two distinct regions that can be used to encode information. The first region has fully aligned spins, while the second region is characterized by a symmetric swirl of spins, akin to a stable MS state. It is plausible to attribute the hard ferromagnetism to the persistent topological order in the spin current because the MS spin texture is created dynamically.

To gain deeper insights to the formation and stabilization of the skyrmions in an external field, it is important to first clarify the role of the Dzyaloshinskii–Moriya interactions (DMI) in the generation and transport of the spin current in multilayers. Thus, the Berry phase theory (BPT) of DMI proposed by Freimuth et al. [86] is adopted in this study. In the BPT, the DMI is the exact ground-state spin current to first order in the spin orbit interaction (SOI). Crucially, the BPT partitions the SOI linear contribution to the DMI into two parts. The first part accounts for the Zeeman interaction between the SOI field and the interfacial spin misalignment acquired by electrons in noncollinear magnetic textures, while the second part arises from the SOI correction to the velocity operator. While the SOI correction to the velocity operator is small in transition metals, its contribution to DMI in HM species cannot be neglected, especially when the HM layer is thick. This is because when the magnetic texture moves in a magnetic multilayer interface, the misalignment of spin carriers leads to a counterpropagating spin current.

A counterpropagating spin current causes the phenomenon of spin backflow, as observed in a related Fe/hBN/graphene/hBN/Pt(Fe) multilayer system [36]. Thus, the spin current driven by magnetization dynamics in Fe(110)/hBN/HM is the injected spin current in the SHE regime, and it carries energy due to the strength of the Zeeman interaction with the SOI field. The related mechanism of inverse designing the driving magnetic fields is used by Yu et al. [87] as the protocol to prepare spin states rapidly and to achieve short-cuts to adiabaticity. Their protocol offers a fast and robust way to control two spins using Heisenberg and Ising interactions. This strategy is used to achieve a fast spin flip, prepare spin states, or generate an entangled Bell state. The fast manipulation of spin states and correction with high fidelity are key requirements for quantum information processing and quantum computing.

From the nature of the observed dynamic transition from the soft to hard magnetic states observed here, it is plausible that the formation of the MS state is due to the presence of geometrical frustration at the interface and the resulting spin transparency. In the Pt-capped stack, the magnitude of the spin memory loss increases with the thickness of the Pt layer in a Fe(110)/Pt(111) bilayer (Figure 3a). Such a sudden switching of spin moments is known to occur in the Ni layer of a FeMn/Ni/Cu(001) multilayer due to interfacial spin frustration. When magnetism in the FeMn overlayer changes from the paramagnetic state to the antiferromagnetic state, it dynamically switches the direction of the ferromagnetic spin moments in the Ni from out-of-plane to in-plane directions of the film. This mechanism only occurs because interfacial spin frustration creates magnetic anisotropy in the Ni film by switching the antiferromagnetic order of the FeMn film [88].

In addition, it is crucial to emphasize that the topological (not the electrical) charge causes the magnetic skyrmion to move with curved trajectories away from the direction of the applied spin current in the skyrmion SHE regime [89]. Thus, even if the skyrmion state is subjected to symmetry-breaking perturbations, the inherent topological charge guarantees its topological protection against external perturbations and thus eliminates the excitation of the topological state. Notwithstanding the stochastic environment of the spins, the intrinsic topological protection guarantees that decoherence cannot occur due to thermal gradient or the effect of an external magnetic field; however, with the low spin flip transition field (i.e., H_sf_ = ~0.5 T), we assert that it is the presence of localized modes that could couple to the skyrmion state that constitutes the main decoherence mechanism. Examples of such localized modes include lattice defects, dislocation modes, spin impurities, dangling bonds, nuclear spins, and localized intramolecular vibrations [90]. This mechanism creates severe constraints on the quality of material design for heterolayer interfaces at the atomic level.

### 3.4. Skyrmion Stabilization in External Fields

The structural evolution and dynamic stabilization of the MS state under an external applied field can be understood at the DFT level by realizing that with an interfacial DMI present between the dissimilar nanomaterials of the stack, the additional Rashba SOC field, which typically exists at surfaces and at interfaces, plays a significant role in the canting of the spin. Thus, the Rashba type spin–orbit splitting in asymmetric Fe(110)/hBN/HM heterostructures causes the spin and momentum degrees of freedom of carriers to lock to each other at the interface. This leads to the conversion of charge current to pure spin current under a SHE regime [18,19]. The direction of the effective Rashba SOC field points in the direction of the spin spiral, i.e., *θ* = 30° relative to the spin quantization axis. Thus, an applied field will tune the skyrmion structure without changing its topological state. Other ab initio calculations have revealed that the related Co/Pt and Fe/Pd/Ir systems exhibit their strongest DMI in the atomic plane of the Co- and Fe-layer that lies closest to the interface [91].

In the Fe(110)/hBN/HM, the additional field due to Rashba SOC points in the direction that plays an important role in the ground state of the multilayer in the presence of a nonvanishing interfacial DMI. This is also due to differences in the density of the spin-polarized charge density [37], which is known to build up between the Fe and *M* (replaced with *HM*) species in such stacks [33]. This charge density difference leads to a non-zero potential difference (Δ*V*) across the constituent heterobilayer interfaces and influences the formation and dynamic stabilization of the MS phase when a spin current exists. Because the electron density profiles of constituent materials of the heterostructure stack are intrinsically different, the redistribution of electron density at the interface during carrier transport induces an electric field in the direction perpendicular to the net magnetization (see Figure 5b). It is physically plausible that this induced electric field breaks the spatial inversion (i.e., z → −z) symmetry and generates an additional non-zero Rashba term. The strength of the Rashba SOC (different for each HM cap) equally determines the magnitude of this induced electric field, and this ultimately determines whether the MS forms or not.

The topological Hall effect (THE) is the abnormal Hall response from the scalar spin chirality of magnetic textures [92]. In the MS state, the skyrmion THE is an equivalent Hall response due to an emergent magnetic field, which is an effect attributable to the Berry skyrmion phase [93]. Thus, within the LLGS formalism of the field-induced skyrmion stabilization considered herein, the spin frustration is attributable to the topological SHE of the chiral spin textures. In both SHE and THE phenomena, formation of the MS and its detection could be manipulated without the need for the STT [94]. By contrast, this is achieved dynamically using the exchange bias. Maccariello et al. [95] recently demonstrated the topological protection of the MS under an anomalous Hall effect (AHE) regime. Recently, Leroux et al. [96] have suggested that the origin of the AHE could be attributed to the step change in the magnetization density **m**, which is introduced in the FM background by the formation of the skyrmion phase. Thus, the change in magnetization density between the ferromagnetic states denoted by Figure 5c and Figure 6c constitutes the dynamic exchange bias required for formation of the MS state in the spin Hall regime.

The dynamic state of the MS therefore responds to the emergent electric field with inherent topological quantization [61,62]. The detection of such a MS in a van der Waals multilayer stack could permit the manipulation of data in memory elements without a STT. Recently, a similar concurrent switching of the ferromagnetic magnetization and exchange bias in the Pt/Co/IrMn trilayer system was obtained by applying current pulses [97]. The MS phase formation illustrated herein is an emergent quantum phenomenon that originates dynamically from the magnetic frustration that arises in the sudden transition from a soft to hard magnetic state when **m** = **p** ≠ 0. With a non-zero topological charge (or winding number), such spin textures are topologically protected against disorder, making them robust carriers of information. The spin frustration, which exists within the FM background, is responsible for the formation of nontrivial soliton phases. Thus, field-induced stabilization of the non-trivial skyrmions is characterized below in the SHE regime.

At the onset of the nucleation of the MS core within the disordered initial spin configuration (Figure 3a), the radius of the MS core changes proportionally with increases in the external magnetic field; however, its phase and topological order show no sensitivity to the external magnetic field. We instead observe a strong influence of the internal exchange fields on the phase and topological order of the MS state. The effective internal field arises as a competition between dominant internal fields in HM-capped heterostructure, which we ascribe to the strong SOC in the HM layer. Its resultant effect on the ferromagnetism contributes to topological frustration in the persistent spin order. At DFT-level by contrast, the geometrical frustration of the spin state arises whenever local spin conditions are incapable of resulting in a simple spin pattern for the extended system [98]. This is expected in the van der Waals ferromagnetic systems due to their heterobilayer interface components.

**Figure 7:**
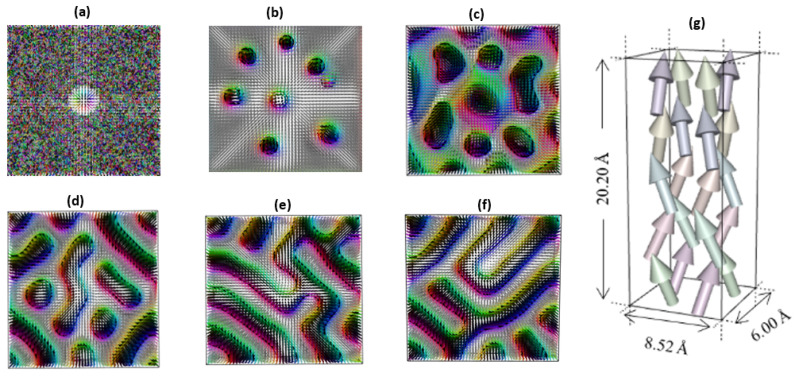
Onset of the nucleation of MS at 25 T (**a**) and its time evolution after 150 ps (**b**). Dynamic stabilization of the skyrmion state at 2.5 (**c**), 0.25 (**d**), 0.025 (**e**), 0.0025 T (**f**), and the unit cell (**g**). These images denote instantaneous results of the spin evolution calculation on a cube containing 30 × 30 × 30 lattice sites. The boxes correspond to 25.56 nm × 18.0 nm × 60.6 nm in size, where the unit cell is a 20-atom box containing 8 Pt and 8 Fe atoms separated by the tunnel barrier region made of a single hBN layer made of 4 atoms, respectively.

Figure 7 shows the field dependence for the skyrmion dynamics. The onset of nucleation of MS at 25 T and at a time evolution after 150 ps is shown in Figure 7a,b, respectively. The time–space spin texture maps reveal that the axes of each of the skyrmions extend asymmetrically into the whole structure; however, the MS axis elongates only in the collinear direction with the applied magnetic field. The thermodynamic limit of the spin textures appears as spherical swirls at 25 T. At this high magnetic field limit, the MS boundaries are sharp, and spin swirls are clearly separated from the ordered spin of the underlying geometry. Such spherical swirls, when coded to the “on state”, in combination with simultaneous coding of the ordered spin background to the “off state”, allows for the bistable control of racetrack memory [99,100]. By contrast, the effect of a continuous reduction in the magnetic field intensity from 25 to 2.5 T is also shown in Figure 7c–f. This reveals that the spin texture exhibits a systemic collapse at low fields. The stable symmetrically spherical swirls observed at 25 T appear to lose their compact shape by continuously coalescing into neighboring spin sites to form curved rod-like spin structures.

Insights to the dynamic stabilization have been obtained from the coexistence of cylindrical and spherical skyrmions (Figure 7c,f). This led to formation of bi-stable skyrmion states at both 2.5 and 0.25 T. Since the merging of nearby skyrmions with locally connected spin sites occurs dynamically in the hard magnetic state, the simultaneous formation of elongated domains of rod-like spin swirls as the MS structure changes in shape is attributable to the interplay of competing fields. A closely related effect has been found to stabilize spin spirals and isolated skyrmions at low magnetic field at vanishing magnetic anisotropy [101]. The effect of a systematic reduction in the applied magnetic field from 2.5 to 0.25 T has been considered. By further reducing the stabilizing field, the rod-like spin swirls at 2.5 T lose their shape (Figure 7d) and form stable MS lines (Figure 7e,f). This creates an interlocking wormhole-like structure as the magnetic field is decreased to 0.025 T. Thus, the spin response to changes in field intensity cannot be attributed to formation of isolated MS domains. The low field strength behavior resembles the formation of MS lines akin to the magnetic vortex lines observed recently in MnSi [102,103].

The magnetic field dependence of the changes in shape suggests that the MS is unstable at low intensities of the external applied field. This agrees with the recent experimental observation of metastable skyrmions in the Co/Ru bilayer when low magnetic fields are applied in the out-of-plane direction [104]. It is therefore plausible that the MS can be displaced at low current densities. Thus, the MS is dynamically stabilized as a secondary phase for carrier transport as a superimposition on the FM state by the applied magnetic field. Our results also agree with recent observations of the displacement of the MS as stable nano-sized magnetic objects by low current densities [103]. Figure 7c–f reveal that the MS has a propensity to move at low de-pinning fields and current densities. This property is promising for spintronic applications because the skyrmion dynamics can be manipulated to bypass lattice defects, thus minimizing energy dissipations through scattering.

### 3.5. Electrical Write-In and Read-Out Operation Cycle

Insights regarding the formation of complex spin domains that possess an inherent MS spin texture in stacked heterolayer nanomaterials are useful for the development of emergent carrier transport and the study of magnetic phenomena [15]; however, to exploit the unique technological advantages of the MS state, active device components must be constructed from suitable magnetic materials and nonmagnetic components. Such materials can deliver the generated MS signal when integrated in logic circuits for electronic devices [105,106]. For instance, skyrmion-based racetrack memory is promising for future data storage technologies, where information can be encoded as binary bits and with nanoscale spin swirls with skyrmions along magnetic strips. Recently, Zhang et al. [107] have reviewed the status of skyrmion electronics with an emphasis on writing, deleting, reading, and processing MSs toward spintronic applications. Read-out operation is crucial for device realizations based on MS states. Although electrical read-out can be achieved using magnetic tunnel junction (MTJ), it is realized herein by harnessing the skyrmion-THE. Spintronic racetrack memory could be made to operate in the same way as the punched tape technology. In this case, a hole represents a “1”, while no hole represents a “0”, such that the presence of a skyrmion would represent a “1” and the absence of a skyrmion would represent a “0” in a way that encodes the original idea. In the following, the possibility of using dynamically switchable magnetic bit to write data to (and read data from) magnetization of the MS state without the need for STT is demonstrated. This could serve as the basis for developing memory technology based on electrical data read-out/write-in operation.

**Figure 8:**
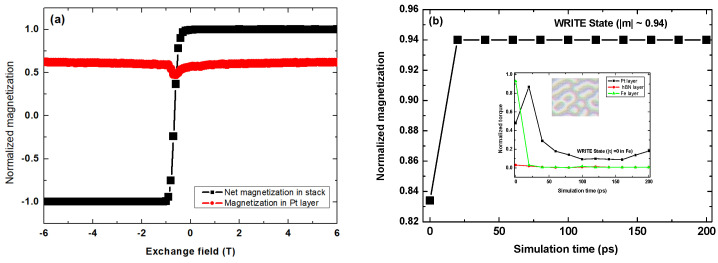
Electrical write-in operation cycle for net magnetization with Pt free layer magnetization at different intensities of the dynamic exchange bias (**a**). Time evolution of the magnetic state of the information vector, showing equilibration at 20 ps and saturation at a normalized magnetization density of ~0.94 within the duration of the write-in pulse (**b**). The microstructure of the corresponding magnetic skyrmion state and asymptotically vanishing layer-resolved spin transfer torques (inset).

Figure 8a shows the cycling of the net magnetization in the memory element and the nonmagnetic Pt layer of the stack at different exchange bias intensities. This shows non-vanishing magnetization in the Pt layer, although the ground state of the electronic structure of Pt shows a nonmagnetic state. Thus, the combination of interfacial spin effects and spontaneous band splitting due to SOC in the presence of the dynamic exchange bias induces non-zero magnetization in the Pt layer. Using the spin current pulse injected via the SHE, data are written either with a forward bias (Figure 6a) or reverse bias (Figure 6b) in terms of the magnetic state. Figure 8b shows the simulation of the normalized magnetization at the start of the write operation and the corresponding skyrmion microstructure. The magnetic state equilibrates after 20 ps and saturates at a normalized magnetization density of ~0.94. This remains stable throughout the 200 ps duration for which the write pulse simulation is implemented. The corresponding layer-resolved normalized torques in the inset of Figure 8b shows systematic drops to zero in both Fe and hBN layers. The magnitude of the torque also approaches zero in the Pt layer. The data writing operation denotes a robust magnetic state of the information vector created by the MS for which a typical storage memory element be based.

**Figure 9:**
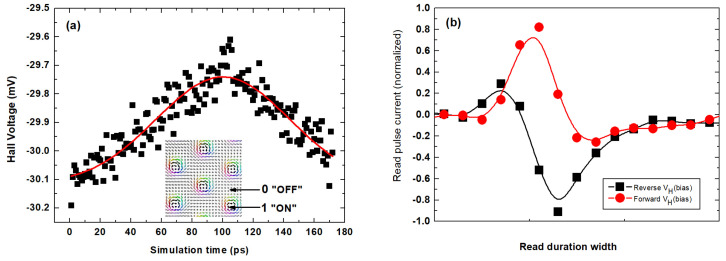
Electrical read-out using the skyrmion-induced Hall voltage (V_H_) signal. The profile of the MS state fitted from the V_H_ spectrum showing nonuniform distribution of spin texture as skyrmion bubbles on the 2D lattice (**a**). The microstructure of the corresponding magnetic skyrmion state at asymptotically vanishing spin transfer torque in the Pt-layer showing binary (0,1) data encoding for the read-out signal (inset). The pulse spectrum of the read-out current for both forward and reverse bias of the maximum V_H_ signal (**b**).

Figure 9 shows the electrical read-out using the skyrmion-induced Hall voltage signal. Figure 9a shows the profile of the MS state fitted from the distribution of the Hall voltages (V_H_) induced by the MS state in a 170 ps duration of the read-in pulse. Clearly, the distribution shows an asymmetrical Gaussian profile due to the intrinsic behavior of spin carriers under the skyrmion with THE. The maximum value of the instantaneous V_H_ is 29.6 mV after 170 ps. It is crucial to emphasize that the negative V_H_ signal (Figure 9a) is a unique contribution of the skyrmion with THE. Its sign is opposite to that of the normal Hall effect, and its magnitude denotes the skyrmion. In a memory cell of constant resistance, the maximum V_H_ signal yields a constant current pulse. For instance, in the racetrack memory element, this constant current is typically ~30 × 10^10^ Am^−2^ for which a duration 0.3 ns is needed to activate the electrical read-in [108]. Figure 9b shows the spectrum of the read-in pulse current for both the forward and reverse bias of the maximum V_H_ signal. The best fit spectrum of the normalized read pulse current shows an inverted signal profile in reverse bias, and nearly constant full width at half maximum (FWHM) independent of the direction of bias. The invariance of the FWHM and the base-to-peak height of both read pulses relative to the constant offset (*I*_0_ = 0) shows the electrical read-in state of the MS within 170 ps.

To develop skyrmion-based logic circuit elements required for racetrack memory cells [88,89], the present study allows the following important inferences to be drawn: (i) Magnetic bits designed to encode binary (i.e., “0” or “1”) data bits correspond to the “on” and “off” states of the read-in/write-out memory operation; (ii) magnetization density in the memory cell is expected to show small jumps in response to repeated V_H_ (Figure 9a) and current pulses of the same polarity (Figure 9b); (iii) the asymmetrical Gaussian profile of the fitted MS state and the corresponding image of the spin microstructure of the 2D lattice of the skyrmion spin texture suggests that the reversal of magnetization in the memory cell is likely to be incomplete. We attribute this partial reversal to the dynamic effect of the SOT field during the SHE; (iv) the nonvanishing torque observed with the Pt layer after 150 ps (insert of Figure 8b) could be caused by the intrinsic SOT but is not entirely clear because Pt is intrinsically nonmagnetic; (v) the nonvanishing magnetization density in the Pt layer of the memory cell (Figure 8a) is therefore a dynamic effect and has no link with the expected Stoner criterion for the quantum mechanical origin of ferromagnetism in Pt or any other HM species; (iv) an externally applied potential bias will change the skyrmion shape to nanoscale spin swirls (Figure 7) without altering the magnetization state of the information vector because the encoded magnetic bits are field-stable.

Thus, once the balance of the internal fields in the device permits the skyrmion state to be present (i.e., state = 1 (on)), the inherent topological protection of the magnetic skyrmion state guarantees that it can only be field-tunable such that the skyrmion is absent (i.e., state = 0 (off)]. This is because neither the on nor off states are excitable by symmetry-breaking fields, since the skyrmion state is topologically protected. As such, the limiting distance for the physical closeness of the bits is naturally related to the decoherence length of the magnetic skyrmion state. Thus, the read/write protocol of one bit is likely to not be influenced by neighboring bits in devices that contain atomically clean interfaces wherein there are no defect states to cause mode coupling; however, when there are localized sources of modes, it is feasible to have their spin textures couple to the skyrmion state to cause bit decoherence.

## 4. Conclusions

In conclusion, first principles calculations have been combined with consideration of the classical dynamics of atomistic spins to demonstrate the formation of robust magnetic skyrmions in frustrated van der Waals magnets modeled as HMs in capped Fe/hBN heterostructures. We have found that the geometrical frustration of spins at interfaces leads to formation of spiral skyrmions with local structure that is tunable by an applied magnetic field. The computed hysteresis loops and magnetic coercivity are analyzed to demonstrate the presence of dynamics exchange bias, which causes metamagnetic transitions characterized by the dynamic switching from soft to hard ferromagnetic state. We find that the skyrmion structure breaks down at low intensity of the applied external magnetic fields. Furthermore, it is shown that the electrical write-in and read-out memory operation is possible using the SHE. These results suggest that the formation of the MS state in the injected spin current density can be used to create dynamically switchable magnetic bits for data storage. These findings are important for spintronic applications because this mechanism could allow data to be written to the soft and hard magnetic state in random-access memory elements without the need for STT.

## Figures and Tables

**Table 1:** Parameters of magnetic exchange interactions, showing the Rashba parameter, uniaxial anisotropy, spin mixing conductance, and Dzyaloshinskii–Moriya and Heisenberg exchange interactions from ab initio calculations.

System	α_R_ (10^−8^ Jm)	*g*^↑↓^ (10^19^ m^−2^)	*K*_u_ (10^6^ Jm^3^)	*J_ij_* (10^−22^ J)	*D_ij_* (10^−22^ J)
Fe(110)/hBN/Pt	4.00	4.9 ± 0.5 [53]	6.6 [54]	28.4	0.61
Fe(110)/hBN/Pd	1.52	2.7 ± 0.4 [55]	1.8 [54]	10.9	0.28
Fe(110)/hBN/Ta	2.77	6.7 ± 0.4 [56,57]	8.1	23.6	0.86

## Data Availability

The data presented in this study are available in the article.
